# 

**Published:** 1882-04

**Authors:** 


					American Medical Association.
The Third Annual Meeting of the American Medical Associ-
ation will be held at St. Paul, Minnesota, commencing Tuesday,
June 6th, and lasting four days. A Section on Dentistry was
formed in this Association at its last meeting, and medically
educated dentists were recognized as specialists in medical
science. All medical men of the regular school, practicing the
specialty of Dental Surgery, are most cordially iuvited to pre-
sent credentials from their local medical societies, and join us
at St. Paul. All rail roads furnish reduced rates to these mem-
bers wishing to attend. "A member desiring to read a paper
Editorial, Etc. 571
before any Section, should forward the paper or its title and
length (not to exceed twenty minutes in reading) to the chair-
man of the Committee of Arrangements, at least one month
before the meeting." (By-Laws.)
Truman W. Brophy,
Sec. Sect, on Dentishy.
Chicago Dental Society.
The annual meeting of the Chicago Dental Society was held
the evening of April 4th, and the following officers were elected
for the ensuing year :
President.?E. S. Talbot.
First Vice President.?Frank H. Gardiner.
Second Vice President.?A. W. Hoyt.
Pec. Secretary.?H. F. Kimball.
Cor. Secretary.?A. W. Harlan.
Treasurer.?E. D. Swain.
Librarian.?Jas. G. Reid.
Board of Directors.?Geo. H. Cushing, J. N. Crouse, Edmund
Noyes.
A. W. Harlan, Cor. Sec.
To Readers and Correspondents.
Communications intended for publication, and exchange journals and pa-
pers, should be addressed to the Editor of the American Journal of Den-
tal Science, 259 N. Eutaw St., Baltimore, Md. All letters relating to the
busineps of the journal, or containing cash remittances, to be directed to
Messrs. Snowden & Cowman. Articles for publication should be received by
the editor at least three weeks previous to the first of the month on which the
number in which it is designed for them to appear, is issued.
ffijfThe American Journal of Dental Science will contain fifty pages
of reading matter in each ntimber, making a volume of about
Six Hundred pages,
EXCLUSIVE OF ADVERTISEMENTS.
T il &
AMERICAN JOURNAL
OF
DENTAL SCIENCE.
The Journal is issued on the first of each month and contains not less
than fifty pages of reading matter in each number, exclusive of adver-
tisements, making a volume of six hundred pages per annum.
In each number will be found Original Communications, Corres-
pondence, Selected Articles, Monthly Summary, Bibliographical No-
tices and Editorial Matter, and every effort will be exerted to render
the Journal worthy of the patronage of the Dental profession.
A generous co-operation is respectfully solicited from the members
of the profession ; &nd as the Journal has now a printing office of its
own, which materially lessens the cost of itspublication.it will be
issued at the reduced subscription price of
SO Per Antrum in Advance^
Communications are respectfully solicited on all subjects pertain-
ing to the practice of dentistry, as well as reports of cases occurring
in dental practice.
HATES OF ADVERTISING.
1 MO.
1 Page.
i " j 10 00
i " I 7 00
$15 00
0 00
7 00
5 00
2 MO.
$22 50
15 00
11 00
8 00
3 MO.
$30 00
20 00
15 00
11 00
6 MO.
$50 00
30 00
20 00
15 00
12 MO.
All original communications designed for publication, works for
review, new instruments for notice, exchanges, &c., should be directed
fo the editor, 259 N. Eutaw St., Baltimore, Md.
All advertisements and subscriptions should be addressed to
SNOWDEN & COWMAN,
Publishers of the Am. Journal of Dental Science.
86 W. Fayette St. Bet. Charles & Liberty Sts.,
BALTIMORE. MI)

				

